# Organic Bio-Based Aerogel from Food Waste: Preparation and Hydrophobization

**DOI:** 10.3390/gels8110691

**Published:** 2022-10-26

**Authors:** Giulia Gaggero, Raman P. Subrahmanyam, Baldur Schroeter, Pavel Gurikov, Marina Delucchi

**Affiliations:** 1 Department of Civil, Chemical and Environmental Engineering, University of Genoa, Via All’Opera Pia 15, 16145 Genoa, Italy; 2 Institute of Thermal Separation Processes, Hamburg University of Technology, Eißendorfer Straße 38, 21073 Hamburg, Germany; 3 Laboratory for Development and Modelling of Novel Nanoporous Materials, Hamburg University of Technology, Eissendorfer Strasse 38, 21073 Hamburg, Germany

**Keywords:** aerogel, bio-based, organic, hydrophobization

## Abstract

In this work, organic aerogels from spent ground coffee and apple pomace were prepared and characterized for the first time. Apple aerogel was found to be much lighter than that from coffee (0.19 vs. 0.016 g/cm^3^, whereas the specific surface areas are comparable (229 vs. 208 m^2^/g). Being intrinsically hydrophilic, these aerogels were silanized, both in liquid and gas phase, to increase stability in aqueous media. The latter modification method allowed chemical grafting of the silane to the aerogel surface (evidenced by FTIR and TGA) and resulted in certain hydrophobicity, as was evidenced via contact angle measurements: both aerogels possess a contact angle of ca. 100° after the gas hydrophobization, while for the pristine aerogels it was 50°. Furthermore, it was observed that the gas-phase silanization process is more applicable to apple aerogels.

## 1. Introduction

Aerogels are lightweight open-pore materials with exceptional properties, i.e., low density, high porosity, and large surface areas [1]. These unique physical properties make them attractive for a wide range of applications from the biomedical and pharmaceutical field, e.g., drug-delivery systems [2] to the environmental field for pollutant removal [3] to the building sector as thermal and acoustic insulators [4,5], as well as for use the food industry [6].

Specifically, silica-based aerogels are extensively studied materials with unique properties, such as high specific surface area (500–1200 m^2^/g), high porosity (80–99.8%), and low density (~0.003–0.5 g/cm^3^). However, the mechanical properties of silica aerogels are usually poor: precursors of synthetic polymer-based aerogels can be toxic and the high cost of preparation have slowed the everyday use of aerogels [7,8].

For this reason, in recent years the development of bio-based aerogels has been of great interest for the scientific community. Other than the previously mentioned physical properties, they are biocompatible and non-toxic. Bio-based aerogels are produced from renewable sources, such as proteins and sugarcane, as well as from biopolymers, such as cellulose, chitosan, and alginate [8,9,10]. Generally, the process of bio-based aerogel preparation involves two steps: formation of a hydrogel and drying. Hydrogels are formed in aqueous media via chemical or physical cross-linking. Considering the drying step, supercritical drying with CO_2_ has been depicted as one of the most effective techniques and requires an additional step of solvent exchange [11,12].

Recently, alongside the use of biopolymers, the concept of organic waste management has gained the attention of scientists where the “reduce, reuse, and recycle” strategy has been awarded. In this context, food waste is a promising raw material for the production of organic or bio-based aerogels. General food waste or by-products from food transformation process are a serious issue for both the economy and the environment. New strategies are required to implement waste and resource management [13,14].

Organic aerogels are a good example of how the scientific community works on sustainable alternatives of traditional processes. Inside this group of materials, it is possible to identify two main branches: recycle-based and organic waste-based aerogels. The basic principle is that they exploit the lignocellulosic content of waste to produce aerogels. For example, textile and paper industries are important sources of cellulosic waste that can be regenerated and used to obtain aerogels [15,16]. Nevertheless, waste biomass valorization is attracting many scientists. banana [17], watermelon [18], and pomelo peel [19] have been used as carbon sources for carbon aerogel preparation as well.

Although research is still at its beginning, waste-based aerogels are a potential solution for developing sustainable nanoporous materials. However, the manufacturing process involves chemicals and energy consumption. An alternative solution to reduce the preparation steps is to use pure biomass, reducing the chemicals and thermal or mechanical processes involved.

Coffee is one of the most consumed brewed beverages in the world. According to the International Coffee Organization ICO, world coffee consumption is projected to increase by 1.9% to 167.58 million bags in 2020/21 compared to 164.43 million bags for 2019/20 [20]. As a consequence of that, there is a high production of solid organic waste. Exhausted coffee or spent ground coffee is a lignocellulosic material mainly disposed of in landfills causing environmental issues [21]. Several recent studies were dedicated to reduce the environmental impact of coffee waste by implementing the cradle-to-cradle design. In fact, coffee has been used in biofuel production, as filler in the construction field, and—because of the presence of nutrients—as fertilizer [22,23]. Furthermore, coffee residues can be further valorized into ecofriendly and multifunctional lignins [24].

Alongside coffee waste management, fruit and vegetable by-products are of great concern for the concept of circular economy. Apple juice production contributes to the development of several tons per year of food waste known as apple pomace [25]. During juice extraction, 75% of fruit weight is pressed and transformed to juice, while the other 25% is pomace. Apple pomace contains pulp, skin, and seeds, and is normally used as animal feed or stock in landfills [26]. However, because of the presence of carbohydrates, proteins, lipids and fibers, several studies have been carried out on the extraction of valuable chemicals, i.e., bakery yeast and pectin, and on the production of biogas out of sugar fermentation [27]. The use of biopolymer aerogels including lignocellulosic materials has been slowed in several application fields due to their intrinsic hydrophilic character. Several attempts have been made to enhance the hydrophobic characteristics, mainly using silane derivatives by chemical vapor deposition(CVD) [28], plasma treatment [29] and fatty acid esterification [30]. However, there is still a lack of simple and cost-effective surface treatments.

The aim of this work was to prepare and characterize organic aerogels from food waste, specifically from spent ground coffee and apple pomace. The main idea is to exploit both their chemical composition and their natural wet state to obtain an aerogel-like morphology, hence the name “organic aerogel.” Keeping in view a potential use of these materials in aqueous media without damaging the porous structure, hydrophilic characteristics were evaluated and modified by means of silanization. Both a liquid phase and a gas phase modification were tested to evaluate the best approach.

## 2. Materials

Ground coffee and apples were purchased from a local supermarket. Ethanol 99.8% was purchased from Sigma Aldrich, Darmstadt, Germany. Tap water was used for the aging test. Methyltrimethoxysilane 97% (MTMS), was purchased from Sigma Aldrich. All chemicals were used without further purification.

### 2.1. Sample Preparation

Coffee aerogels, CA, were prepared as follows. Wet spent ground coffee powder was rinsed with hot water until the supernatant was light brown. Solvent exchange was carried out by immersing the samples in ethanol/water mixtures increasing the ethanol concentration, i.e., to 60, 90, 100 wt.% of ethanol. The concentration of ethanol was controlled by density meter (DMA 4500, Anton Paar Company, Austria). When the final ethanol concentration exceeded 98 wt.%, samples were transferred to supercritical CO_2_ drying [31].

Apple aerogels, AA, were prepared by directly mixing apple pomace, without seeds, with anhydrous ethanol. Solvent exchange and supercritical drying were carried out following the same procedure as used for coffee.

### 2.2. Surface Modification

Silanes are well-known hydrophobic agents for cellulose. They can form polysiloxane structures by reacting with a hydroxyl group of the cellulose fibers through a condensation reaction [32,33]. Surface modification of both coffee and apple aerogels was carried out by silanization in either liquid or gas phase.

The liquid phase method, LPM, involved the samples before supercritical drying, thus at the end of the solvent exchange phase. The prepared samples, known as alcogels, were filtered from pure ethanol to obtain 0.5 g of wet solid and immersed in 20 mL of a 5 wt.%. MTMS solution in ethanol 80 wt.% for 3 h at 60 °C. All samples were washed with ethanol to remove the non-reacted silane.

The gas phase method, GPM, is based on the chemical vapor deposition technique. Around 0.5 g of organic based aerogel was placed in a 100 mL bottle together with two small, opened vessels containing 2 mL of MTMS and 1 mL of deionized water, respectively. The bottle was then sealed and placed in an oven at 80 °C for 5 h. After the reaction time, the bottle was quickly removed from the oven and opened under a laboratory hood to remove the excess of silane. Samples were named HCA_(l)_ and HAA_(l)_ to indicate the hydrophobic modification of the aerogels in liquid phase, while HCA_(g)_ and HAA_(g)_ indicate the hydrophobic modification in gas phase.

## 3. Characterization Techniques

Microstructure of the samples was studied by scanning electron microscopy (Leo Gemini 1530, Zeiss, Oberkochen, Germany). Samples were sputtered with a thin layer of gold (approx. 6 nm, Sputter Coater SCD 050, BAL-TEC) to increase the conductivity. Measurements were carried out at an accelerating voltage of 4 kV and under high vacuum.

Bulk density, ρ_b_ (g/mL), was determined using a graduated cylinder. Dry aerogels were poured into the cylinder up to a certain volume and weighted. The bulk density was calculated as a ratio of the particles weight and the occupied volume.

The water vapor uptake of the samples was determined at 20 °C by keeping the samples in a climate chamber with 80% relative humidity (RH) for 72 h. The water adsorption percentage was calculated according to Equation (1).

(1) $$V_{u}\;\left(\%\right)=\frac{w_{t}-w_{d}}{w_{d}}\times 100\%$$

where $V_{u}$ is the percentage of absorbed water vapor, $w_{t}$ is the wet weight after 72 h, $w_{d}$ is the dry weight of the samples.

Determination of the specific surface area was carried out by low-temperature N_2_ adsorption analysis (NOVA 4000e, Quantachrome Instrument. Anton Paar, Graz, Austria) using the Brunauer-Emmett-Teller (BET) method.

The hydrophilic/hydrophobic characteristics of aerogels were determined via contact angle measurements using the static sessile drop method. Around 0.2 g of powder were pressed to form a pellet and a waterdrop of 15 µL was dripped onto its surface. The drop profile was allowed to stabilize before measurement.

IR spectra were recorded at room temperature in the range 400–4000 cm^−1^ with a Bruker Vertex 70 instrument equipped with attenuated total reflectance (ATR) accessory with a resolution of 2 cm^−1^.

Thermogravimetric analysis, TGA, was performed with a STA PT 1600 Linseis to investigate the thermal stability between 30 °C and 600 °C at a heating rate of 10 °C/min in a nitrogen atmosphere.

## 4. Results and Discussion

### 4.1. Morphological Analysis

**Figure 1:**
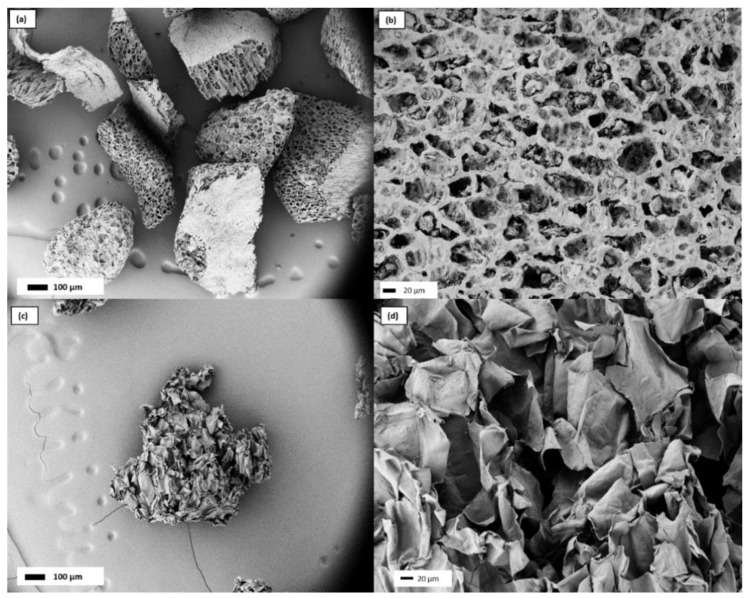
SEM micrographs of coffee aerogels at (**a**) 100× magnification and (**b**) 500× magnification. SEM micrographs of apple aerogels at (**c**) 100× magnification and (**d**) 500× magnification.

Scanning electron microscopy was used to investigate the microstructure as well as macroscopic size and shape of coffee and apple aerogel particles (Figure 1).

The production process resulted in aspherical aerogel particles with a size of approx. 300–400 µm for both CA and AA.

Morphologically, the coffee aerogel shows a compact honeycomb-like structure with irregular macropores of 20–30 μm in diameter that apparently confers a high toughness to the aerogel. By increasing the magnification, it is possible to see smaller cavities inside the pores of around 1 μm in diameter (Figure 1b). The apple aerogel appears, instead, like an aggregate of flakes randomly crumpled with a wide space among them (Figure 1c,d). In this case, the system seems to be crumbly.

**Figure 2:**
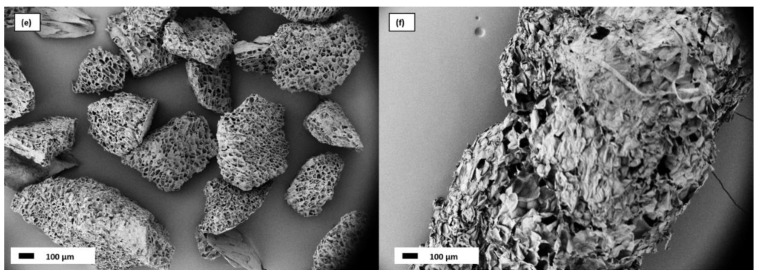
SEM micrographs at 100× magnification of: (**e**) coffee aerogels after water immersion test, (**f**) apple aerogels after water immersion test.

The effect of water on the morphology and physical properties was investigated by immersing the aerogels in water for 24 h and letting them dry under ambient conditions. In Figure 2, SEM micrographs of CA (e) and AA (f) after the water aging process are shown.

CA macroporous structure seems not to be affected by water, while AA shows a collapse of the structure, as seen by the compaction of the flakes and the loss of voids among the sheets.

### 4.2. Physical Properties

Table 1 shows the bulk density and BET surface results of pristine and modified aerogels.

Considering the S_BET_ results, both pristine CA and AA have a comparable high specific surface area of about 200 m^2^/g. Considering the different morphology, this result can be justified with a high amount of mesopores in the CA that compensates its apparently more compact structure with respect to the one of AA.

The results show that both LPM and GPM reduce the surface area, whereas HCA_(l)_ and HAA_(l)_ have a higher surface area compared to HCA_(g)_ and HAA_(g)_. On the one hand, the reduction in S_BET_ after LPM can be ascribed to the deposition of MTMS that partially obstructs the micropores. On the other hand, the dramatic decrease in specific surface area that can be observed in the samples after GPM may be due to silane deposition and the effect of water vapor and temperature promoting the collapse of the microstructure. The difference that can be observed between HCA_(g)_ and HAA_(g)_ is probably rooted in the higher hydrophilicity of coffee aerogel than apple aerogel, which induces stronger interactions between the fibers of the cellulose with consequent shrinkage of the system.

Table 1 shows the bulk density of coffee and apple aerogels. Specifically, CA has a higher bulk density then AA. Because coffee- and apple pomace-derived aerogels have never been reported, a direct comparison with other existing materials is not possible. However, both aerogels have a bulk density comparable with traditional lightweight porous materials [8]. The very high difference between the samples is correlated with the different shape of the particles that induced a different packing inside the cylinder used for the bulk density measurement. In fact, the apple aerogel flakes can be packed more loosely than the coffee aerogel. Moreover, it is interesting to notice that CA bulk density is only slightly affected by LPM, while after GPM, an increased value of ρ_b_ is detected. Furthermore, apple aerogels showed an increased ρ_b_ after both liquid and gas phase modification. An increase in bulk density after silanization can be linked to the deposition of silane on the aerogel surface. Moreover, the absence of humidity between the particles enhances the packing ability and flowability of particles, avoiding the formation of sticky large aggregates, thus increasing the bulk density. Considering this hypothesis, surface modification worked better for apple then coffee. Moreover, because of the higher bulk density, gas phase modification seems to be more effective as a modification technique.

### 4.3. Water Vapor Uptake

The influence of air humidity on the aerogel particles was tested at 80% RH. Water vapor uptake is an important parameter that affects degradation and induces changes in mechanical properties. This test is a useful tool to evaluate the effectiveness of hydrophobic modification. The results are reported in Table 2.

By weighing the samples before and after the humidity exposure, pristine aerogels, CA and AA, have a humidity absorption of 13 and 16%, respectively. With the LPM, the humidity absorption increases to 15% for HCA_(l)_, and 22% for HAA_(l)_. On the contrary, samples modified by GPM exhibit an uptake of 13% for HCA_(g)_, and 12% for HAA_(g)_. This means that chemical vapor deposition has no effect on coffee, while it leads to better results for apple aerogels.

### 4.4. Wetting Ability

Table 3 shows the wettability results for pristine and modified aerogels. To better appreciate the hydrophilic/hydrophobic nature of the materials, two pictures were taken, at 5 s and 60 s after the waterdrop was deposited on the surface.

According to Table 3, CA, AA, and their modification with LPM demonstrate immediately a small contact angle; however, after 60 s, all liquid was soaked by the sample, making measurements impossible. In contrast, the samples treated with the GPM demonstrated good water repellence by keeping a constant high contact angle after 60 s. The results are reported in Table 4.

### 4.5. ATR-FTIR Spectroscopy

**Figure 3:**
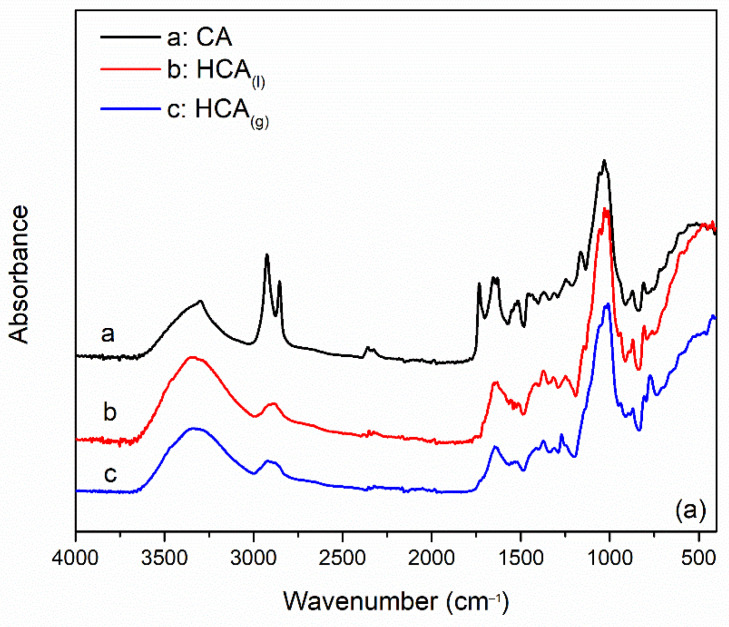

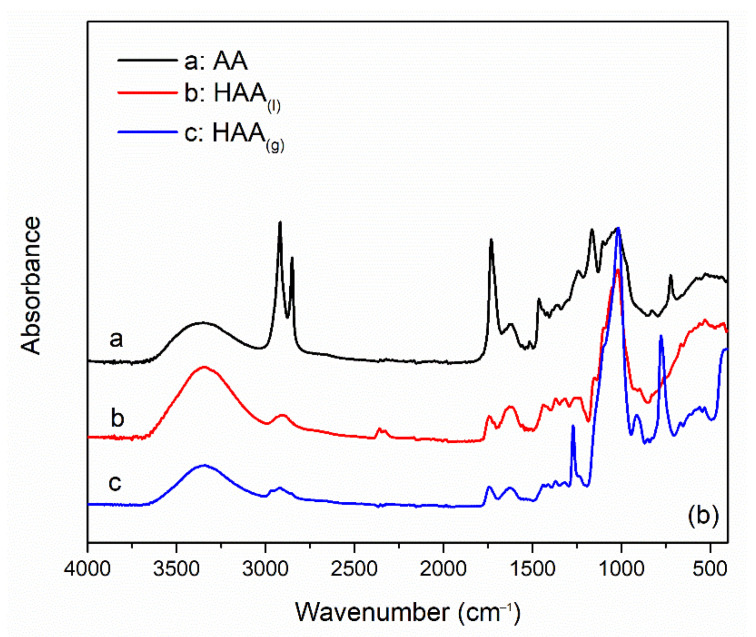
FTIR spectra collected in ATR mode of pristine aerogel (black curves), after LPM silanization (red curves), and after GPM silanization (blue curves). (**a**) CA, HCA_(l)_ and HCA_(g)_. (**b**) AA, HAA_(l)_ and HAA_(g)_.

The chemical composition and the interaction between aerogels and silane were investigated by ATR-FTIR spectroscopy. Figure 3 shows the absorbance spectra of the pristine and silanized aerogels.

FTIR spectra of all the coffee aerogels (Figure 3a) show a broad peak between 3500 and 3000 cm^−1^ related to the hydroxyl group of O-H stretching vibration. Considering the CA curve, the most representative peaks at 2925 cm^−1^ and at 2855 cm^−1^ correspond to the C-H stretching vibration of cellulose backbone. The signal at 1733 cm^−1^ is related to the C=O from carbonyl group in aliphatic esters. The small peak at 1655 cm^−1^ represents the carbonyl stretching from lignin moieties [34]. The wide peak at 1100–1300 cm^−1^ may be assigned to the C-C vibration of cellulose [21]. What is interesting to notice for both HCA_(l)_ and HCA_(g)_, is that the broad band O-H stretching vibration band is shifted at 3351 cm^−1^. Considering HCA_(l)_, the two peaks at 2925 cm^−1^ and 2855 cm^−1^ are merged in a single peak with a lower absorbance that can be caused by the presence of Si-CH_3_ bond. Other characteristic peaks cannot be detected because of the strong overlap between the two spectra. HCA_(g)_ showed some significant differences compared to CA, the bending of the Si-CH_3_ was observed with a peak at 1270 cm^−1^, and the absorption bands related to the stretching vibrations of the Si–C bond and/or the stretching vibrations of the Si–O bond were observed at 776 cm^−1^ [35].

In the IR spectrum of apple aerogels, Figure 3b, the most representative peaks are associated to O-H stretching broad band at 3354 cm^−1^, to the asymmetric and symmetric stretching vibration of C-H at 2918 and 2850 cm^−1^, respectively, and to the characteristic vibration of carboxyl group C=O at 1731 cm^−1^. A few complex bands in the range of 1000–1200 cm^−1^ are due to C-O and C=C stretching modes of the organic chain [25,36]. Considering HAA_(l)_ and HAA_(g)_, O-H characteristic peak is slightly shifted at 3340 cm^−1^, the peaks at 2924 and 2854 are merged in a single smaller peak at 2911 cm^−1^, the peak associated with the carboxyl group is shifted as well to 1746 cm^−1^. Again, due to the overlapping in the region between 1200 and 1000 cm^−1^, it is difficult to determine the characteristic peak of Si-O-Si. However, it is possible to identify the peak associated with the bending vibration of Si-CH_3_ bond at 1250 cm^−1^ for HAA_(l)_ and at 1271 cm^−1^ for HAA_(g)_. Moreover, the latter exhibit specific peaks at 913 cm^−1^ attributed to Si–OH bond stretching vibrations, and at 777 cm^−1^ related to the absorption bands of the stretching vibrations of the Si–C bond and/or the stretching vibrations of the Si–O bond [37,38].

The results confirmed the presence of silica species in the aerogels. However, gas phase modification showed a higher degree of functionalization with respect to coffee aerogels.

### 4.6. Thermal Stability

**Figure 4:**
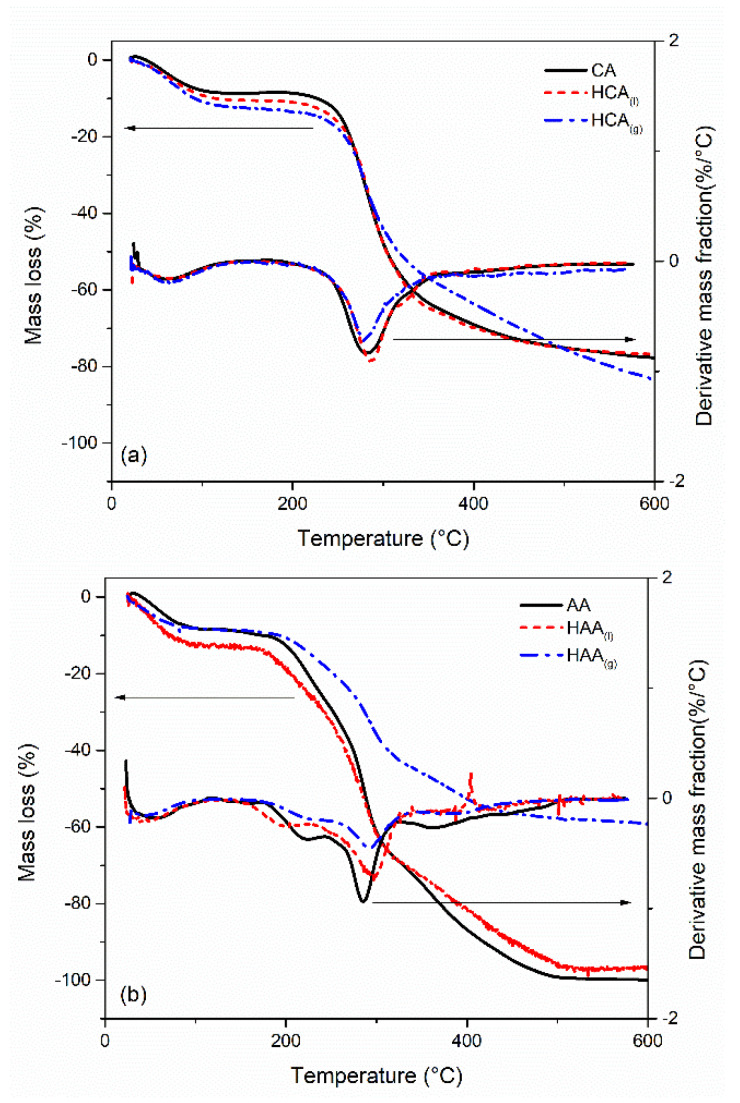
Thermal stability investigation. TGA and DTG profiles of pristine aerogel (black curves), LPM silanization (red curves), and GPM silanization (blue curves). (**a**) CA, pristine and after LPM and GPM treatment, (**b**) AA, pristine and after LPM and GPM treatment.

The thermal stability of all the samples was evaluated by thermogravimetric assessment. Surface modification was investigated qualitatively. TGA and DTG curves are shown in Figure 4.

Coffee aerogel curves show two clear degradation steps. The first one between 80 and 100 °C is the loss of moisture. The second one occurs between 200 and 400 °C, and is caused by the degradation of lignin and cellulose [39,40]. However, at the end of the thermal program, around 20% of the total mass remains as a solid residue. Considering the modified aerogel, HCA_(l)_ has an almost identical curve, meaning that no additional silicon dioxide can be detected. Compared to HCA_(l)_, HCA_(g)_ shows a lower slope between 300 and 500 °C. This behavior can be related to an alteration in the structure during the GPM technique. From the DTG curves (derivative mass fraction %), two-step degradation is confirmed. The maximum of each peak describes the point of greatest rate of change on the weight-loss curve. Two broad peaks with, respectively, a maximum at 65 and 281 °C are depicted for CA. However, the maximum of the peak associated with the lignocellulosic degradation shifted slightly to 288 °C and 279 °C for HCA_(l)_ and HCA_(g)_, respectively.

The thermal decomposition of apple aerogels (Figure 4b) occurs in four steps. The first step ends around 100 °C and can be attributed to moisture loss. The three degradation steps occurring between 200 and 500 °C are characteristic of the lignocellulosic compounds [36]. According to previous works, the weight loss that occurs at 200 °C describes the degradation of lignin and hemicellulose. Between 250 °C and 300 °C pyrolysis of cellulose also starts, whereas the pyrolysis process is completed at approx. 500 °C [41,42]. This behavior is confirmed by the presence of four peaks in DTG curves at 40, 222, 285 and 362 °C, respectively. Considering HAA_(l)_, few differences with respect to the pristine AA are depicted: the thermal stability slightly improved after 300 °C with a positive shift of the maximum correlated with the lignocellulosic degradation. However, no modification of hydrophobic characteristics can be detected. On the other hand, HAA_(g)_ shows not only a better thermal stability all over the curve but also a higher solid residual. These results evidence the presence of (CH_3_)_3_Si groups on the surface of apple aerogel.

## 5. Conclusions

Organic waste-based aerogels were prepared using spent ground coffee and apple pomace. To the best of our knowledge, this publication is the first report on aerogels from these origins. Pristine coffee and apple aerogels exhibited a comparatively high surface area and low bulk density. Their morphology was studied by SEM: coffee aerogels showed a honeycomb-like structure, while apple ones resembled a messy folded sheet structure. As evidenced by SEM, apple aerogels undergo a collapse when immersed in water. In order to use these materials in aqueous media, two surface treatments by means of silanization were carried out: a liquid phase modification, where MTMS was added to an ethanol solution, and a gas phase modification, which involved pure MTMS in the vapor phase. CA modification was not entirely successful in either liquid or gas phase, as shown by the bulk density, FTIR, and TGA results. On the other hand, probably because of the different morphology and chemical structure, the silanization increased the hydrophobic characteristics of apple aerogels, as confirmed by the bulk density, wetting ability, FTIR, and TGA results. This work may open new avenues for the utilization of biopolymer waste streams by converting them into porous materials with added value.

## Figures and Tables

**Table 1:** Physical characterization of pristine coffee and apple aerogels (CA and AA), and after hydrophobic surface modification (HCA and HAA). n.d. means not detectable.

	S_BET_ (m^2^/g)		ρ (g/cm^3^)	
Treatment/Sample	CA	AA	CA	AA
Pristine	229 ± 20	208 ± 20	0.191 ± 0.004	0.016 ± 0.001
After LPM	152 ± 20	75 ± 20	0.207 ± 0.001	0.026 ± 0.001
After GPM	n.d.	11 ± 20	0.265 ± 0.001	0.032 ± 0.001

**Table 2:** Water vapor uptake after 72 h in a controlled humidity chamber at RH 80%.

	Water Absorption (%)	
Treatment/Sample	CA	AA
Pristine	13.4 ± 0.9	15.6 ± 0.4
After LPM	14.8 ± 0.6	22 ± 3
After GPM	14 ± 2	12 ± 1

**Table 3:** Investigation of surface wettability. Contact angle measurement after 5 and 60 s of pristine aerogels and after silanization in liquid phase (LPM), and in gas phase (GPM).

Treatment/Sample	CA 5 s 60 s	AA 5 s 60 s
Pristine	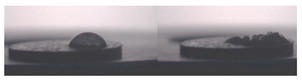	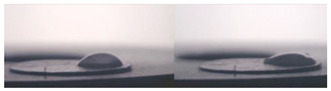
After LPM	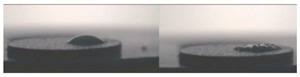	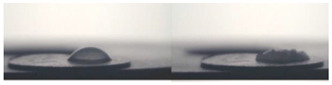
After GPM	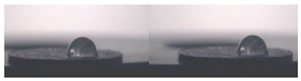	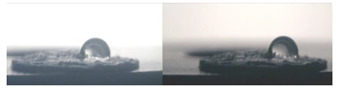

**Table 4:** Contact angle θ for pristine aerogels and after silanization in liquid phase (LPM) and in gas phase (GPM) at t = 0 s and after 60 s. n.d. means not detectable.

	θ_5s_ (°)	θ_60s_ (°)	θ_5s_ (°)	θ_60s_ (°)
Treatment/Sample	CA		AA	
Pristine	50 ± 2	n.d.	50 ± 2	n.d.
After LPM	43 ± 3	n.d.	49 ± 2	n.d.
After GPM	97 ± 3	97 ± 3	100 ± 3	100 ± 3

## Data Availability

The data presented in this study are available on request from the corresponding author.
